# Association of late‐life cognition and brain pathology with anti‐hypertensive medications

**DOI:** 10.1002/alz.092438

**Published:** 2025-01-09

**Authors:** Ajay Sood, Ana W. Capuano, Rupal I. Mehta, Lisa L. Barnes, David A. Bennett, Zoe Arvanitakis

**Affiliations:** ^1^ Rush University, Chicago, IL USA; ^2^ Rush University Medical Center, Chicago, IL USA; ^3^ Rush Alzheimer’s Disease Center, Chicago, IL USA; ^4^ Rush Alzheimer’s Disease Center, Rush University Medical Center, Chicago, IL USA; ^5^ Department of Psychiatry and Behavioral Sciences, Rush University Medical Center, Chicago, IL USA; ^6^ Department of Neurological Sciences, Rush University Medical Center, Chicago, IL USA; ^7^ Rush Medical College, Chicago, IL USA; ^8^ Department of Neurological Sciences, Rush Medical College, Chicago, IL USA

## Abstract

**Background:**

Hypertension is a risk factor for cognitive impairment and dementia. Anti‐hypertensives (AHT) are commonly used in old age, but their association with cognition and brain pathology is not well understood.

**Method:**

To investigate the relation of AHT with change in cognitive function and postmortem brain pathology, we evaluated 4,207 older persons without known dementia at enrollment and a subset of 1880 participants who died and came to autopsy. Participants underwent yearly clinical evaluations that included an assessment of medications and neuropsychological testing across five cognitive domains. The participants who died underwent neuropathologic evaluation of neurodegenerative (amyloid, Lewy bodies, Tar DNA binding protein ‐43, hippocampal sclerosis) and cerebrovascular pathology (infarcts, amyloid angiopathy, atherosclerosis, or arteriosclerosis).

**Result:**

Over 3,600 participants were using AHT, of which 64% were using diuretics, 55% beta‐blockers, 50% calcium channel blockers, 44% angiotensin‐converting enzyme inhibitors, and 29% angiotensin receptor blockers any time during the study. Using a linear mixed effect model adjusted for age, sex, race and education, AHT users at study entry had a higher level (estimate = 0.106, SE = 0.025, p <0.001) and a slower decline of global cognition compared to non‐users (estimate = 0.013, SE = 0.013, p = 0.013). Analyses of cognitive domains showed a similar pattern with a higher level at study entry and a slower decline of semantic memory (estimate = 0.025, SE = 0.007, p<0.001). The use of AHT was not associated with change in other domains. In a separate analysis including only participants with neuropathology, AHT use was associated with lower tau tangle density (estimate = ‐0.20, SE = 0.07, p = 0.005) after adjusting for age at death, sex, and race. The use of AHT was not associated with other neurodegenerative pathologies or cerebrovascular pathology.

**Conclusion: Conclusions:**

AHT use in old age is associated with slower cognitive decline and fewer tau tangles in the brain. Future research will need to replicate these findings and explore underlying mechanisms by which AHTs appear to have beneficial effects on cognition.

This work is supported by the following NIH grants: P30AG10161, P30AG072975, R01AG15819, R01AG17917, 5R01NS084965, 1RF1AG059621, 1RF1AG074549, R01 AG22018.

